# Area under the Curve-Based Dosing of Vancomycin in Critically Ill Patients Using 6-Hour Urine Creatinine Clearance Measurement

**DOI:** 10.1155/2020/8831138

**Published:** 2020-12-24

**Authors:** Bita Shahrami, Farhad Najmeddin, Saeideh Ghaffari, Atabak Najafi, Mohammad Reza Rouini, Mojtaba Mojtahedzadeh

**Affiliations:** ^1^Department of Clinical Pharmacy, Tehran University of Medical Sciences, Tehran, Iran; ^2^School of Pharmacy, Tehran University of Medical Sciences, Tehran, Iran; ^3^Department of Anesthesiology and Critical Care, Tehran University of Medical Sciences, Tehran, Iran; ^4^Department of Pharmaceutics, Tehran University of Medical Sciences, Tehran, Iran

## Abstract

**Background:**

The area under the curve- (AUC-) guided vancomycin dosing is the best strategy for individualized therapy in critical illnesses. Since AUC can be calculated directly using drug clearance (CL_van_), any parameter estimating CL_van_ will be able to achieve the goal of 24-hour AUC (AUC_24 h_). The present study was aimed to determine CL_van_ based on 6-hour urine creatinine clearance measurement in critically ill patients with normal renal function.

**Method:**

23 adult critically ill patients with an estimated glomerular filtration rate (eGFR) ≥60 mL/min who received vancomycin infusion were enrolled in this pilot study. Vancomycin pharmacokinetic parameters were determined for each patient using serum concentration data and a one-compartment model provided by MONOLIX software using stochastic approximation expectation-maximization (SAEM) algorithm. Correlation of CL_van_ with the measured creatinine clearance in 6-hour urine collection (CL_6 h_) and estimated creatinine clearance by the Cockcroft–Gault formula (CL_CG_) was investigated.

**Results:**

Data analysis revealed that CL_6 h_ had a stronger correlation with CL_van_ rather than CL_CG_ (*r* = 0.823 vs. 0.594; *p* < 0.001 vs. 0.003). The relationship between CL_van_ and CL_6 h_ was utilized to develop the following equation for estimating CL_van_: CL_van_ (mL/min) = ─137.4 + CL_6 h_ (mL/min) + 2.5 IBW (kg) (*R*^*2*^ = 0.826, *p* < 0.001). Regarding the described model, the following equation can be used to calculate the empirical dose of vancomycin for achieving the therapeutic goals in critically ill patients without renal impairment: total daily dose of vancomycin (mg) = (─137.4CL_6-h_ (mL/min) + 2.5 IBW (kg)) × 0.06 AUC_24 h_ (mg.hr/L).

**Conclusion:**

For AUC estimation, CL_van_ can be obtained by collecting urine in a 6-hour period with good approximation in critically ill patients with normal renal function.

## 1. Introduction

Vancomycin is still used as the standard treatment for suspected methicillin-resistant *Staphylococcus aureus* (MRSA) infections in intensive care units (ICUs). This feature has dramatically increased the utilization of vancomycin despite the introduction of some alternative agents [1,2]. Because of the narrow therapeutic range of vancomycin, therapeutic drug monitoring (TDM) is an important issue for achieving optimal levels of this antibiotic particularly among patients with critically ill conditions [3].

According to the vancomycin dosing guidelines, the area under the curve (AUC) is the best predictor for drug dosing [4]. Evidence shows the supratherapeutic threshold of vancomycin in trough levels between 15 and 20 mg/L as recommended before, and dose adjustment based on trough level alone is not an accurate estimation of 24-hour AUC (AUC_24 h_) [5–7]. AUC-guided dosing is associated with a lower risk of nephrotoxicity due to reducing the irrational overuse of vancomycin [8, 9]. Based on this, converting from the trough level to AUC has occurred for the vancomycin's therapeutic goals, and the AUC_24 h_ target of 400−600 mg·hr/L is recommended regardless of minimum inhibitory concentration (MIC) or treated organism [10, 11].

Since vancomycin AUC is estimated by drug clearance, if there is a way to predict the clearance, the AUC_24 h_ will be easily calculated in the patients. The main route for clearance of vancomycin in the body is almost exclusively through the kidneys. Nearly most of the vancomycin is recovered unchanged in urine through glomerular filtration [12, 13]. If the patient's kidney condition is accurately estimated, then a better decision will be made on the dose of vancomycin; furthermore, accumulation of vancomycin can cause serious side effects, including nephrotoxicity and ototoxicity [14]. The best marker for determining renal function is the glomerular filtration rate (GFR) [15]. There are several methods for measuring GFR, among which creatinine clearance (CrCL) is used based on urine collection [16, 17]. Previous studies reported some positive aspects regarding using creatinine measurement such as easy measurement, as well as not being invasive and expensive [18]. Also, it is endogenous and does not need to be injected. Accordingly, CrCL is used as the clinical surrogate for GFR.

Currently, the guidance on the vancomycin dosing is based on the CrCL obtained from the Cockcroft–Gault (CG) equation. Since the ICU patients have unstable conditions, application of CG equation to adjust drug dosing may lead to ending up with subtherapeutic and supratherapeutic trough concentrations [19]. This equation does not consider increasing the cardiac output and renal flow rate due to hemodynamic changes and medications used in the ICU, thus underestimating CrCL or overestimating the degree of acute kidney injury (AKI) in critically ill patients [20]. As a result, assessment of renal function presents a unique challenge in critical illnesses, and it should be noted that none of the existing methods are capable of truly predicting the clearance of vancomycin (CL_van_). Therefore, there is a need to introduce new methods or optimize existing ones. Hence, the current study was aimed to determine CL_van_ for AUC estimation based on 6-hour urine creatinine clearance (CL_6 h_) measurement in critically ill patients with normal renal function.

## 2. Materials and Methods

### 2.1. Study Settings

This prospective pilot study was approved by the Institutional Ethics Committee of Tehran University of Medical Sciences (TUMS), Iran.

### 2.2. Patient Selection

23 critically ill patients aged older than 18 years with a normal renal function (estimated glomerular filtration rate (eGFR) ≥60 mL/min) who received vancomycin infusion for treatment were enrolled in the study after obtaining the informed consent. None of the patients had known hypersensitivity to vancomycin, and all patients had started using this drug by attending the physicians for the treatment of presumed or documented Gram-positive infections. Patients with unstable kidney function during 48 hours before and after the first dose were excluded from the study [21]. All patients initially received a loading dose of 25 mg/kg of vancomycin based on total body weight and with an infusion period of ≥30 minutes for every 500 mg, followed by an intermittent infusion.

### 2.3. Methods to Collect and Measure Vancomycin Clearance

Serum concentrations of vancomycin were collected from a central venous (CV) line. Peak and trough levels were drawn 1-hour after the end of the infusion and 1-hour before the next dose infusion, respectively. After centrifugation, all plasma samples were analyzed by fluorescence polarization immunoassay through EMIT assays (Siemens Healthcare Diagnosis, United Kingdom, EMIT).

First-dose pharmacokinetics was performed for each individual patient using the one-compartment model. Pharmacokinetic parameters (such as CL_van_ and AUC_24 h_) were calculated by MONOLIX software as the mean of their posterior distribution using stochastic approximation expectation-maximization (SAEM) algorithm.

Renal function was monitored for all patients using serum creatinine and urine output. CrCL was estimated using the CG formula.

(1) $$\begin{array}{c} {\text{CL}}_{\text{CG}}\;=\frac{\left(140-\text{AGE}\right)\;\times \text{ IBW}}{72\;\times \text{ SCr}}\times 0.85\;\left(\text{if female}\right)\text{,} \end{array}$$

where CL_CG_ is the Cockcroft–Gault creatinine clearance (mL/min), AGE is the age of the patients (years), IBW is the ideal body weight (kg), and SCr is the serum creatinine (mg/dL).

In addition, a 6-hour urine collection was performed for measuring urinary creatinine concentration and urine volume for all patients. Then, CL_6 h_ can be determined as follows:

(2) $$\begin{array}{c} {\text{CL}}_{6\text{ h}}=\frac{V_{u}\;\times C_{\text{uCr}}}{T\;\times \;C_{\text{sCr}}}, \end{array}$$

where CL_6 h_ is the 6-hour urine creatinine clearance (mL/min), *V*_*u*_ is the urine volume (mL), C_uCr_ is the urine creatinine concentration (mg/dL), *T* is the duration of urine collection (minutes), equivalent to 3600 minutes for our patients, and C_sCr_ is the creatinine serum concentration (mg/dL).

### 2.4. Statistical Analysis

Correlation of CL_van_ with the measured CrCL from the 6-hour urine collection (CL_6 h_) and the estimated CrCL (CL_CG_) was investigated by the Pearson correlation test, and the final model was achieved by linear regression analysis within a stepwise protocol. Data are presented as mean (95% confidence interval) or median (1^st^ quarterly and 3^rd^ quarterly), as appropriate. All the collected data were analyzed by SPSS software version 25. For all tests, *p* value <0.05 was considered as statistically significant.

## 3. Results

The subjects of the study were the patients admitted to the general and emergency ICU wards of Sina Hospital affiliated to TUMS. A majority of the patients were male (81%) with an average age of 45.1 ± 19.4 years old. The mean Acute Physiology and Chronic Health Evaluation II (APACHE II) score was 12.1 ± 1.5. Regarding the body weight, total body weight (TBW) was 71.1 ± 14.8 kg, and ideal body weight (IBW) was 68.4 ± 9.7 kg. The median (1^st^ quarterly and 3^rd^ quarterly) daily dose of vancomycin therapy was equal to 46.9 [42.9, 48.6] mg/kg in patients. The pharmacokinetic parameters of vancomycin studied in patients are listed in Table 1.

**Figure 1:**
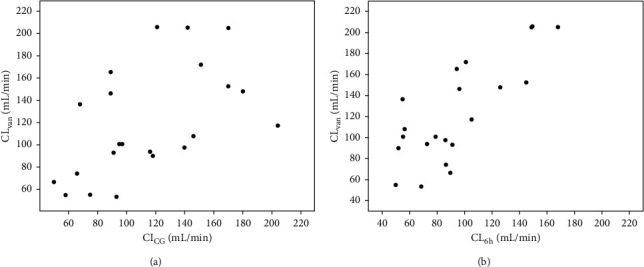
Comparisons between CL_van_, CL_6 h_, and CL_CG_. (a) Correlation between CL_van_ and CL_CG_. (b) Correlation between CL_van_ and CL_6 h_. Abbeviations: CL_van_: clearance of vancomycin, CL_6 h_: measured creatinine clearance in 6-hour urine collection, CL_CG_: estimated creatinine clearance by the Cockcroft–Gault formula.

**Figure 2:**
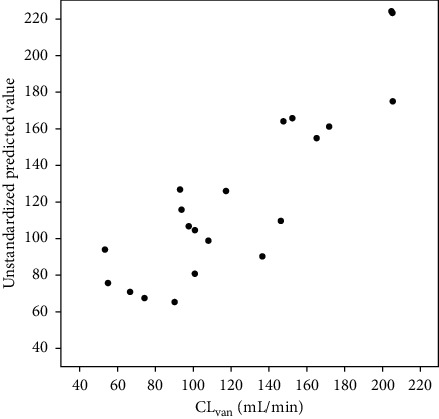
Correlation between predicted values of CL_van_ and actual values. Abbeviations: CL_van_: clearance of vancomycin.

CL_6 h_ was significantly closer to CL_van_ than CL_CG_ (*r* = 0.823 vs. 0.594; *p* < 0.001 vs. 0.003). The results of comparisons between these parameters are shown in Figures 1 and 2.

Given that the AUC depends on the clearance, different parameters were included in the regression model to find the most accurate prediction model for CL_van_. CL_CG,_ CL_6 h_, TBW, IBW, and age were tested. CL_6 h_ and IBW were found to be the best predictors for the model. The final equation was identified with respect to the best predictive ability to estimate CL_van_ in critically ill patients without renal impairment as follows:

(3) $$\begin{array}{c} {\text{CL}}_{\text{van}}\left(\text{mL}/\min\right)=-137.4+{\text{CL}}_{6\text{ h}}\left(\text{mL}/\min\right)+2.5\text{IBW}\left(\text{kg}\right)\;\left(R2=0.826,p<0.001\right), \end{array}$$

Regarding the described model, the practitioners can use the proposed equation to calculate the empirical dose of vancomycin to achieve the AUC_24 h_ goal. For this purpose, the following equation can be used:

(4) $$\begin{array}{c} \text{total daily dose of vancomycin }\left(\text{mg}\right)={\text{CL}}_{\text{van}}\left(\text{mL}/\min\right)\times {\text{AUC}}_{24\text{ h}}\left(\text{mg}\cdot \text{hr}/\text{L}\right). \end{array}$$

Accordingly, AUC_24 h_ may be considered between 400 and 600 mg·hr/L based on the targeted MIC with a goal of AUC_24 h_/MIC ≥400; as vancomycin MIC for sensitive *Staphylococcus aureus*, *Staphylococcus epidermidis*, and *Enterococcus* spp. ranges between 1 and1.5 mg/L,

(5) $$\begin{array}{c} \text{total daily dose of vancomycin }\left(\text{mg}\right)=\left(-137.4+\right.{\text{CL}}_{6\text{ h}}\left(\text{mL}/\min\right)+2.5\text{IBW}\left(\text{kg}\right)\\ \times 0.06\times {\text{AUC}}_{24\text{ h}}\left.\left(\text{mg}\cdot \text{hr}/\text{L}\right)\right). \end{array}$$

Table 2 could be simply used to determine the appropriate total daily dose of vancomycin based on measured CL_6 h_.

## 4. Discussion

AUC-guided dosing strategy as the critical target value for individualized vancomycin dosing can be estimated using several methods including the Bayesian approach, two-point sampling, and continuous infusion [22]. Bayesian dose-optimizing software used for estimating AUC working by merging a single level of vancomycin with the populations' pharmacokinetics needs to be purchased, and many health systems, particularly in developing countries, have no access to this program [23]. Pharmacokinetic equations based on two or more vancomycin levels provide good accuracy with less bias, but it is associated with the increased laboratory workload and health-care costs [24]. Considering the limitations of current methods for AUC estimation of vancomycin, the present study was conducted to propose a new tool for achieving the therapeutic goals of vancomycin therapy and could be considered as an alternative method for empirical dosing when the lab data are available.

In case of critical illnesses, equations used for estimating renal function including CG formula are not able to provide correct judgment because this formula is based on the kidney status of the healthy men, and factors such as muscle mass, excess fat, or fluid in obese subjects and secretion of creatinine from renal tubules are not considered in this formula [6, 25]. Moreover, the CG equation is insensitive and is not capable of showing abrupt and acute changes in kidney condition since changes in the serum creatinine status do not occur quickly [26, 27]. Besides, this formula is no longer recommended for estimating vancomycin clearance and consequently for maintenance dosing's [28, 29]. According to the results of a current systematic review [30], using urinary CrCL is the best diagnostic method for estimating drug dosing in augmented renal clearance (ARC). 24-hour urine collection is often difficult, and some limitations are reducing the accuracy of collecting urine within 24 hours [31, 32]. In case of the critical condition of the ICU, requiring quick decision-making urine collection can be implemented by decreasing the time of urine collection. Several studies have been conducted to compare the results of 24-hour urine collection with those collected in less duration, and it has been concluded that 24-hour urine collection can be replaced with urine collection in less time [32–34]. Nevertheless, few of them have compared the urine CrCL with drug clearance such as vancomycin, which is almost completely removed from the kidneys. Rodvold et al. included a larger sample of 37 patients into three groups based on measured 24-hour CrCL including patients with renal impairment. The resulting equation (CL_van_ (mL/min/1.73 m^2^) = 15.7 + 0.79 CL_24 h_ (mL/min/1.73 m^2^)) produces similar results to the equation derived in this study [35]. Zokufa et al. have suggested that renal biomarkers measurement (e.g., cystatin C) may be used to estimate CrCL and to determine the vancomycin dosing in ICU patients [36].

To our knowledge, this is the first research using 6-hour urine collection in ICU and comparing it with CL_van_ and CG equation in patients with normal renal function and including ARC. The 6-hour urine collection was selected as urine collection in our ICU is performed every 3 hours under the supervision of nurses, and the 6-hour duration only involves the participation of one nurse in this process and may reduce the errors. In this study, a strong relationship was found between CL_van_ and CL_6 h_. Therefore, a new modeling trend was determined for vancomycin dosing based on urine collection as an alternative method for empirical initiation while waiting for serum level measurements.

### 4.1. Study Limitations

Since this research was a single-center pilot study, the described model should be confirmed by further studies with large multicenter data to evaluate vancomycin AUC_24 h_ based on 6-hour urine CrCL measurement in ICU patients. The described model only fits the patients with an eGFR ≥60, and this could be considered as another important limitation of this study. On the other hand, as shown in the plots, this model may fit accurately in patients with ARC. Since the sample size of this study was small, we could not perform multiple adjustments for any other independent variables. However, we included one variable for every ten patients (1 : 10) into the multiple linear regression model to have enough power.

## 5. Conclusions

Considering the cost and labor intensity related to applying the TDM process, the results of the present study revealed that, for AUC estimation, CL_van_ can be obtained by collecting urine in a 6-hour period with good approximation in critically ill patients with normal renal function.

## Figures and Tables

**Table 1:** Pharmacokinetic parameters of vancomycin.

Measures	Mean ± S.D. or median (1^st^ quarterly and 3^rd^ quarterly)
*t*_1/2_ (hr)	5.7 [5.2, 7.0]
*V*_*d*_/kg (L/kg)	0.75 ± 0.23
CL_van_ (mL/min)	96.2 ± 29.9
AUC_24 h_ (mg.hr/L)	518 [447, 641]

Abbreviations: S.D.: standard deviation, *C*_*p*_: peak concentration, *C*_*t*_: trough concentration, *t*_1/2_: half-life, *V*_d_/kg: volume of distribution per kilogram, CL_van_: clearance of vancomycin, and AUC_24 h_: 24-hour area under the curve.

**Table 2:** Appropriate total daily dose of vancomycin in various ranges of CL_6 h_ to achieve target AUC_24 h_ of 400 or 600 mg·hr/L considering IBW equal to 70 kg.

CL_6 h_ (mL/min)	A total daily dose of vancomycin (mg) to achieve target AUC_24 h_	
	Target AUC_24 h_ of 400 (mg·hr/L)	Target AUC_24 h_ of 600 (mg·hr/L)
60	2250	3500
80	2750	4000
100	3250	4750^*∗*^
120	3750	─

^*∗*^Target AUC_24 h_ of 400 mg.hr/L may be considered because of the total daily dose >4 g. Abbreviations: CL_6 h_: measured creatinine clearance in 6-hour urine collection; AUC_24 h_: 24-hour area under the curve.

## Data Availability

The figure and table data used to support the findings of this study are included within the article.
